# Negative Temperature Dependence of Recrystallized Grain Size: Formulation and Experimental Confirmation on Copper

**DOI:** 10.3390/ma10030308

**Published:** 2017-03-21

**Authors:** Mohamed Elmasry, Fan Liu, Yao Jiang, Ze Ning Mao, Ying Liu, Jing Tao Wang

**Affiliations:** School of Material Science and Engineering, Nanjing University of Science and Technology, Nanjing 210094, China; masrawy1975@yahoo.com (M.E.); liufan_njust@126.com (F.L.); jiangyaonjust@126.com (Y.J.); maozening@163.com (Z.N.M.)

**Keywords:** catalyzed nucleation, high angle grain boundary (HAGB), recrystallization, equal channel angular pressing (ECAP), negative temperature dependence

## Abstract

The catalyzing effect on nucleation of recrystallization from existing grains resulting from previous lower temperature deformation is analyzed, analogous to the size effect of foreign nucleus in heterogeneous nucleation. Analytical formulation of the effective nucleation site for recrystallization leads to a negative temperature dependence of recrystallized grain size of metals. Non-isochronal annealing—where annealing time is set just enough for the completion of recrystallization at different temperatures—is conducted on pure copper after severe plastic deformation. More homogeneous and smaller grains are obtained at higher annealing temperature. The good fit between analytical and experimental results unveils the intrinsic feature of this negative temperature dependence of recrystallized grain size.

## 1. Introduction

The history of metalworking—including the procedures of deformation and heating—can be traced back to the early days of the Neolithic Age, when the first metalsmiths began working with native copper [1]. The birth of “recrystallization” was declared by a paper of 1887, where Sobey reported the emergence of new equiaxed grain when heating the deformed iron with elongated structure [2]. Establishing quantitative models based on the physics of recrystallization and using them to improve, optimize, and control microstructure has long been the crucial mission of the modern metal industry, and also the frontier of nanomaterials research [3].

In summarizing the “laws of recrystallization”, it was stated by Burke and Turnbull [4,5] that the final grain size obtained from recrystallization by annealing after deformation depends chiefly on the degree of deformation, and to a lesser degree on the annealing temperature—normally being smaller the greater the degree of deformation and the lower the annealing temperature. Experimental observations show that final grain size increases significantly with the increase of annealing temperature after pre-deformation in steel [6,7], Cu–Zn alloys [8,9], magnesium alloy [10], molybdenum alloy [11], etc. However, on the other side, Eastwood et al. reported that recrystallized grain size is insensitive to annealing temperature and disclosed that different annealing temperatures yield a similar grain size after recrystallization for brass [12].

In addition to these contradictory experimental results, a basic gap in our knowledge for many years has been the lack of a quantitative model to account for the grain size after recrystallization [13]. One principal reason for the existence of this basic gap is the complexity of the recrystallization process—especially the complex nucleation behavior of pre-deformed materials. In order to reach a quantitative formulation of the recrystallized grain size, and to simultaneously avoid the complex problem related to the nucleation process, it will be helpful to quantitatively estimate the number of effective nucleation sites (which acts as a nucleus when dynamic conditions are satisfied during the nucleation process), which serves as an essential base for calculating the nucleation rate for recrystallization, and is always proportional to the final number of recrystallized grains after the completion of recrystallization.

The existing high angle grain boundaries (HAGB) in pre-deformed materials play an important role in promoting nucleation for recrystallization, through mechanisms such as strain-induced boundary migration (SIBM) [14]. Severe plastic deformation introduces a high density of non-equilibrium HAGBs, which serves as preferential nucleation site for recrystallization during annealing subsequent to the deformation, and significantly promotes the nucleation rate which leads to a much smaller recrystallized grain [15,16]. Analogous to the catalyzed nucleation by “foreign particles” during phase transformation, the catalyzing effect on nucleation of recrystallization from HAGB can be analyzed by following the work on foreign nuclei in heterogeneous nucleation [17,18].

## 2. Analytical Formulation of Catalyzed Nucleation by HAGB in Recrystallization

Consider the catalyzing effectiveness on nucleation of recrystallization from an existing grain with a diameter of *d* in the as-deformed microstructure with HAGBs, where the catalyzed embryo has a critical radius of r*, which can be calculated from the classical nucleation theory [17]:

(1) $$r^{*}=\frac{2\gamma }{\Delta G_{V}},$$

where *γ* is the interfacial energy and Δ*G_V_* is the difference of Gibbs free energies per unit volume of the parent and new phases; in the present case of recrystallization, this Gibbs free energy difference equals to stored energy from pre-deformation, and is basically constant upon change of annealing temperature.

Fletcher [17] showed that the free energy of formation of a critical embryo with a radius of r* can be written as

(2) $$\Delta G^{*}=\frac{8\pi \gamma^{3}}{3{\left(\Delta G_{V}\right)}^{2}}f\left(m,x\right),$$

where *f*(*m*, *x*) is known as the Fletcher shape factor for heterogeneous nucleation on a convex spherical catalyst particle, and can be given as

(3) $$f\left(m,x\right)=1+{\left(\frac{1-mx}{g}\right)}^{3}+x^{3}\left[2-3\left(\frac{x-m}{g}\right)+{\left(\frac{x-m}{g}\right)}^{3}\right]+3mx^{2}\left(\frac{x-m}{g}-1\right),$$

where *g* = (1 + *x*^2^ − 2*mx*)^1/2^; *x* = *d*/(2r*) is the dimensionless diameter of the spherical catalyst particle, normalized by the radius of critical embryo r*, and *d* is the actual dimeter of the catalyst particle; *m*—in the case of melt solidification—is the cosine of the contact angle of the new embryo on the catalyst surface, with 0 ≤ *m* ≤ 1. In the present case of recrystallization, *m* resembles the catalyzing efficiency of the pre-existing grain boundary on the formation of a new embryo for recrystallization, and could be considered constant in the case of high angle grain boundary misorientation. Thus, *f*(*m*, *x*) is a function depending solely on *x*, the diameter of existing grains with HAGBs catalyzing the nucleation of recrystallization in subsequent annealing.

Generally, in dealing with the effect of foreign particle size on heterogeneous nucleation [19] and the nucleation issue of a pre-deformed material during recrystallization, probability has been used to relate nucleation activities with structure parameters in the parent phases (such as grain boundary misorientation, etc.) [13,20]. Hence, the probability *p* for an existing grain in the pre-deformed matrix to act as an effective nucleation site for recrystallization is considered. It is reasonable to assume *p* to be proportional to a thermal activation factor with ΔG* as the energy barrier, and *f_HAGB_* the fraction of HAGBs which are evidenced to be preferential nucleation sites. Thus,

(4) $$p\approx C\exp\left(-\frac{\Delta G^{*}}{k_{B}T}\right)f_{HAGB},$$

where *C* is a coefficient, *T* is the absolute temperature of annealing, and *k_B_* is the Boltzmann constant.

In considering the catalyzed nucleation by “foreign particles” during phase transformation, it is clear that the larger the diameter of the “foreign particle”, the higher its efficiency in catalyzing the nucleation. Analogous to this tendency, suppose *p* ≈ 1 at *x* ≥ *x*_c_, which means grains in the pre-deformed matrix with HAGB and normalized dimensionless diameter *x* larger than *x*_c_ will act as effective nucleation sites upon annealing at temperature *T* with 100% possibility; and considering Equations (2) and (3),

$$p\approx 1\approx C\exp\left[-\frac{8\pi \gamma^{3}}{3\Delta G^{2}{}_{V}k_{B}T}f\left(m,x_{c}\right)\right]f_{HAGB}$$

This leads to a relation between the annealing temperature *T* and pre-existing grain size *x*_c_:

(5) $$\frac{8\pi \gamma^{3}}{3\Delta G^{2}{}_{V}k_{B}T}\;f\left(m,x_{c}\right)=Tln\left(Cf_{HAGB}\right),$$

Fletcher [17] showed that shape factor *f*(*m*, *x*_c_) increases monotonically upon decrease of *x*_c_, and Equation (5) gives a mathematically proportional relationship between *f*(*m*, *x*_c_) and *T*; thus, the higher the *T*, the larger the *f*(*m*, *x*_c_), and the smaller the *x*_c_, a drastic decrease of *x*_c_ upon *T* is thus resulted.

**Figure 1:**
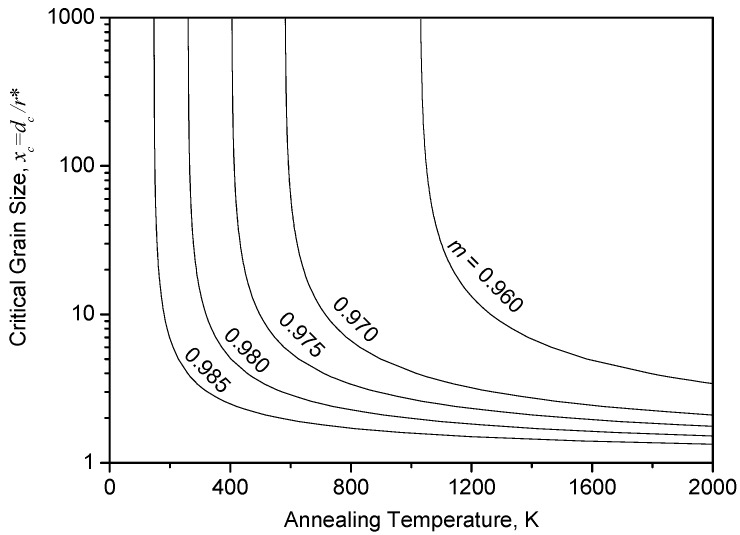
Dependence of critical grain size *x*_c_ upon annealing temperature *T* at different levels of catalyzing efficiency *m*, which is large enough to sufficiently catalyze nucleation of recrystallization.

Using typical parameters of *f_HAGB_* = 0.67 [21], Δ*G_V_* = 57 J/mol ≈ 8 × 10^6^ J/m^3^ [22] after severe plastic deformation, *γ* = 0.625 J/m^2^ [2] for copper, and *C* = 1.51, Equation (5) gives analytical results as shown in Figure 1. All the curves describing the dependence of *x*_c_ upon *T* at different catalyzing efficiency *m* indicate that the higher the annealing temperature *T*, the smaller the critical grain size *x*_c_, and thus more grains in the pre-deformed matrix will act as effective nucleation sites. This means that higher annealing temperatures lead to smaller recrystallized grain size or a negative temperature dependence of recrystallized grain size, which is not only against the “laws of recrystallization” summarized by Burke and Turnbull [4,5], but also contradictory to the experimental results for copper [23,24,25,26,27], as well as other metals and alloys [6,7,8,9,10,11].

In studying the recrystallization behavior of a two-phase brass, a negative temperature dependence of recrystallized grain size was observed by Naether et al. [9], where the reduction in the average grain size with increasing temperature was observed, accompanied by a concurrent increase of the volume fraction of *β* phase in the sample. This case did not indicate the intrinsic negative temperature dependence of recrystallized grain size itself, but the increasing effect of *β* phase stabilization from the concurrent increase of the volume fraction of *β* phase in the sample upon increase of annealing temperature [9].

The key to unlocking this contradiction between the analytical formulation given in Equations (2)–(5) and the experimental data in the literature might be the annealing time. Isochronal annealing at different temperatures is usually used for the annealing and recrystallization to obtain temperature dependence of grain size in the literature [23,24,25,26,27]. When the annealing time is set to be enough for completion of recrystallization at a lower temperature, this isochronal annealing time is far more than that for recrystallization to complete at higher temperatures, and the grain size obtained in this case for high temperature annealing inevitably includes the effect of grain growth subsequent to the completion of recrystallization, and this leads to the resulting “pseudo-morph” of positive temperature dependence of grain size after “recrystallization”.

To avoid the influence of this isochronal annealing issue and to obtain a true annealing temperature dependence of recrystallized grain size, kinetics of recrystallization processes could be characterized first, and the time just enough for the completion of recrystallization at each annealing temperature could thus be determined, which could then be used for non-isochronal annealing at different temperatures to obtain grain size immediately after the completion of recrystallization process. This non-isochronal annealing may remove the effect of grain growth after the completion of recrystallization, and obtain the intrinsic annealing temperature dependence of recrystallized grain size.

## 3. Experimental Confirmation

To confirm the negative temperature dependence of recrystallized grain size unveiled by the above analytical formulation, cold rolled (CR) high purity copper (99.97 wt %) after equal channel angular pressing (ECAP) was used.

**Figure 2:**
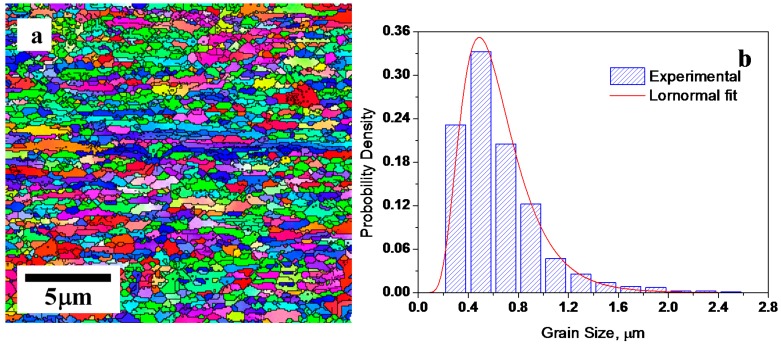
An electron back-scattering diffraction (EBSD) image of (**a**) the cross-section and (**b**) its grain size distribution of high purity (99.97 wt %) copper pre-deformed by equal channel angular pressing (ECAP) + cold rolling (CR).

Hot rolled high purity copper with initial heat treatment at 873 K (600 °C) for 1 h to achieve a homogenous microstructure with an average grain size of ~100 μm was used in this study. Billets with size of 32 × 32 × 160 mm^3^ were processed by ECAP at room temperature with a die angle of 90° for eight passes using route Bc [28]. The sample was then cold rolled at room temperature to a thickness reduction of ~75%. After pre-deformation through ECAP + CR, annealing was conducted at temperatures ranging from 373 K (100 °C) to 573 K (300 °C) for times ranging from 3 to 48,000 s. Vickers microhardness was measured at room temperature with a load of 100 g for 10 s. More than 20 random indentations were made to obtain a representative bulk hardness value. Electron back-scattering diffraction (EBSD) technique was used to characterize microstructural evolution on the cross-sections of the ECAP + CR samples during annealing treatment, and grain boundaries with a misorientation angle ≥15° were considered HAGBs. Figure 2 shows the EBSD microstructure of the sample after cold rolling (Figure 2a) and its grain size distribution (Figure 2b). The HAGB fraction of the materials was ~68%.

**Figure 3:**
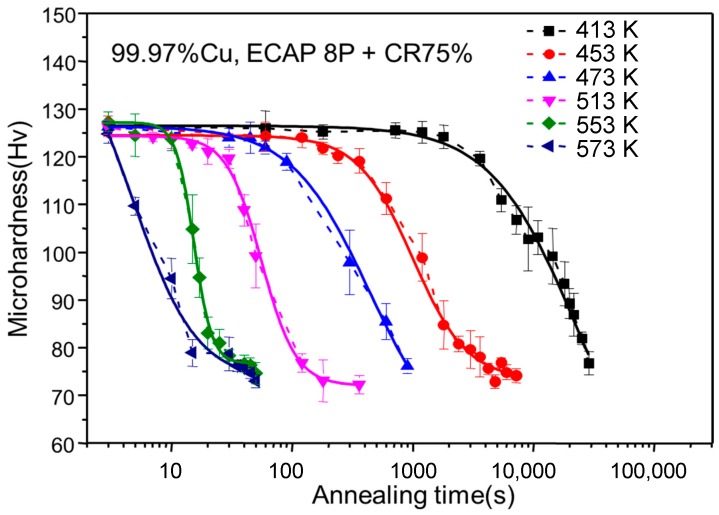
Variation of the microhardness vs. time at different annealing temperatures for ECAP + CR copper samples.

The recrystallization kinetics of the ECAP + CR samples upon annealing was characterized by the microhardness variations versus annealing time for different isothermal annealing temperatures, as shown in Figure 3, which indicates that when the annealing temperature increased from 413 K (140 °C) to 573 K (300 °C), the time for the completion of the recrystallization was reduced from 8 h to 35 s.

**Figure 4:**
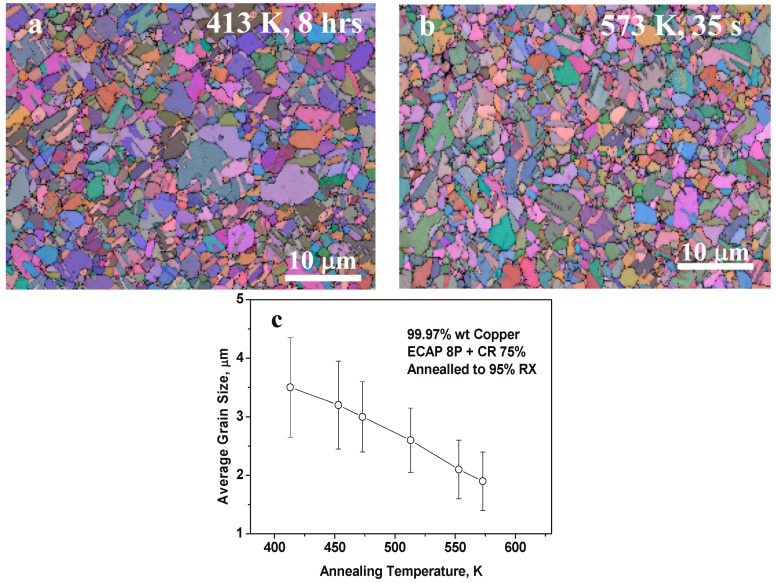
EBSD images after ~95% recrystallization of the specimens upon annealing at (**a**) 413 K (140 °C), 8 h; (**b**) 573 K (300 °C), 35 s; and (**c**) the dependence of the recrystallized grain size *D*_T_ at 95% recrystallization (RX) on annealing temperature.

Figure 4a,b shows the microstructure of the fully (~95% completion) recrystallized specimens annealed at 413 K (140 °C) and 573 K (300 °C) respectively. The recrystallized grain size after 413 K/8 h annealing ranges from 1 to 15 µm with an average grain size of 3.5 ± 0.9 µm, while that after the 573 K/35 s annealing ranges from 1 to 7.5 µm with an average value of about 1.9 ± 0.5 µm.

The microstructures clearly show that the microstructure becomes more homogeneous with smaller average grain size with increasing annealing temperature, which means that the recrystallized grain size has a negative dependence on the annealing temperature. Grain size data from specimens annealed at other temperatures also confirm this negative dependence, as shown in Figure 4c. It is clear from this figure that the recrystallized grain size at ~95% completion of recrystallization decreases, and even the distribution span of grain size at each annealing temperature also decreases upon the increase of annealing temperature.

To compare these experimental results with the prediction of Equation (5), grain size distribution in the as-deformed matrix is needed. It has been demonstrated that the grain size *d* distribution after severe plastic deformation follows the log normal distribution [29,30].

(6) $$f\left(d;\mu ,\sigma \right)=\frac{1}{d\sigma \sqrt{2\pi }}\exp\left[-\frac{{\left(lnd-\mu \right)}^{2}}{2\sigma^{2}}\right],$$

where the two distribution parameters *μ* and *σ* are the mean and standard deviation of ln*d*, respectively. The number fraction of grains with a diameter ≥*d*_c_ (*d*_c_ = 2 r*·x_c_) is:

(7) $$F_{N}=1-\int_{0}^{dc}f\left(x;\mu ,\sigma \right)dx\;=\frac{1}{2}-\frac{1}{2}erf\left(\frac{lnd_{c}-\mu }{\sigma \sqrt{2}}\right),$$

On the other hand, from the experimental data, the number of grains in the recrystallized microstructure annealed at temperature *T* is inversely proportional to its average grain size *D_T_*, and the number of grains in the pre-deformed matrix is inversely proportional to its average grain size *d_av_*. Thus, the number fraction of grains in the pre-deformed matrix which transformed into recrystallized grains after annealing at temperature *T* is:

*F_NT_* = (*d_av_*/*D_T_*)^3^. (8)

Combination of Equations (7) and (8) gives:

(9) $$erf\left(\frac{lnd_{c}-\mu }{\sigma \sqrt{2}}\right)=1-2\left(\frac{d_{av}}{D_{T}}\right),$$

**Figure 5:**
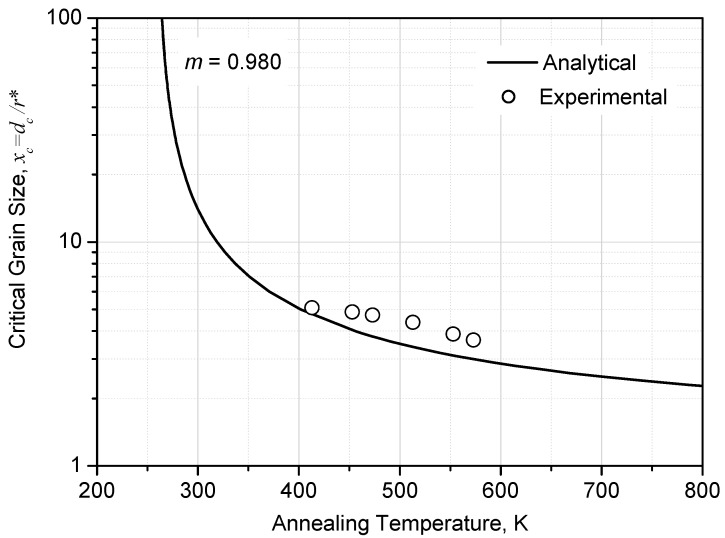
Comparison of analytically and experimentally obtained dependence of critical grain size for catalyzing nucleation site (*x*_c_ = *d*_c_/2r*) upon annealing temperature *T*.

*d_av_* can be calculated from Figure 2b, together with *μ* = −0.538 and *σ* = 0.424 for the lognormal distribution of grain size, and *D_T_* is given in Figure 4c. Thus, *F_NT_* and *d*_c_ for recrystallized microstructure annealed at temperature *T* can be calculated according to Equations (8) and (9). An experimental relation of *x*_c_ (*x*_c_ = *d*_c_/(2r*)) and *T* can thus be obtained, and is shown in Figure 5 by hollow circles. The curve for *m* = 0.980 from Figure 1—which is an analytical prediction from Equation (5)—is also plotted in Figure 5. It can be seen that the data points from experiments fit fairly well with the analytical prediction for *m* = 0.980. The high value of *m* (=0.980)—which yields a good fit between analytical prediction and experimental data—reflects the high catalyzing efficiency of the pre-existing HAGB on the forming of new embryo for recrystallization.

## 4. Conclusions

In summary, the catalyzing effect on nucleation of recrystallization from pre-existing grains with HAGB is analyzed, analogous to the size effect of foreign nucleus in heterogeneous nucleation. Analytical formulation of effective nucleation sites leads to a negative temperature dependence of recrystallized grain size, where a critical grain size *d*_c_ pre-existing in the as-deformed matrix is formulated to be negatively dependent on the annealing temperature *T*; grains with diameter larger than this critical *d*_c_ in the as-deformed matrix will act as effective nucleation sites for recrystallization in the subsequent annealing at *T*. The higher the *T*, the smaller the *d*_c_, and the higher the fraction of grains in the as-deformed matrix can act as effective nucleation sites, and thus smaller recrystallized grain size is formulated. Non-isochronal annealing—where annealing time is set just enough for the completion of recrystallization at different temperatures—is conducted on a high purity (99.97 wt. %) copper after room temperature severe plastic deformation. Negative temperature dependence of recrystallized grain size is confirmed, where more homogeneous and smaller grains are experimentally obtained at higher annealing temperature. The good fit between analytical prediction and experimental results unveils the intrinsic feature of the negative temperature dependence of recrystallized grain size.
